# Pulmonary artery banding in infants and young children with end-stage left ventricular dilated cardiomyopathy: cohort study

**DOI:** 10.1097/JS9.0000000000002061

**Published:** 2024-08-26

**Authors:** Min Zeng, Fan Yang, Chao Yue, Wei Wei, Kai Ma, Zheng Dou, Quanlin Li, Xu Wang, Shoujun Li

**Affiliations:** a Department of Pediatric Cardiac Center, National Center for Cardiovascular Diseases, Fuwai Hospital, Chinese Academy of Medical Sciences and Peking Union Medical College; b Department of Pediatric Intensive Care Unit, National Center for Cardiovascular Diseases, Fuwai Hospital, Chinese Academy of Medical Sciences and Peking Union Medical College; c Department of Pediatric Cardiac Surgery Center, National Center for Cardiovascular Diseases, Fuwai Hospital, Chinese Academy of Medical Sciences and Peking Union Medical College, Beijing, People’s Republic of China

**Keywords:** dilated cardiomyopathy, heart failure, infant, medication therapy, pulmonary artery banding

## Abstract

**Background:**

Dilated cardiomyopathy (DCM) is the most common cardiomyopathy, and 40-–50% of patients may die or need a heart transplant in 5 years after diagnosis. Although heart transplantation is the most effective life-saving option of end-stage DCM, scarcity of donors and series of complications prevent many patients from receiving timely treatment. Pulmonary artery banding (PAB) is recently described as an alternative strategy for end-stage DCM, with low left ventricular function (LVEF) but preserved right ventricular function, may potentially restore heart function and delay the need for heart transplantation, but current clinical evidence is still insufficient. On the other hand, the medication treatment of DCM in pediatric patients is mostly based on the experience of adults. It remains unclear whether PAB combined medication treatment could benefit infants and young children patients. The aim of this study was to assess the short-term efficacy of PAB combined with medication therapy in infants and young children with end-stage DCM, compared with medication therapy alone.

**Methods:**

This is a retrospective analysis of 18 consecutive pediatric patients aged ranging from 1 month to 44 months old who diagnosed with end-stage DCM (LVEF ≤30%) with preserved right ventricular function between 2019 and 2023 in our hospital. All patients had been treated with conventional medications for 2 months. Then they were divided in two groups: PAB surgery group (6/18), and nonsurgery group (12/18). Regardless of whether surgery was performed, both groups continued to receive medication treatment. Recovery of ventricular function was primary endpoints. Secondary endpoints included 180-day mortality and severe heart failure (LVEF ≤30%).

**Results:**

The authors found there were no differences in age, weight, height, BMI, renal function, liver function, pulmonary hypertension, tricuspid valve regurgitation, mitral valve regurgitation, and genetic abnormalities between those with and without PAB surgery. Comparing with nonsurgery group, five patients in surgery group regain the normal cardiac ejection fraction (LVEF ≥50%) (5/6, 83.3% vs. 4/12, 33.3%, *P=*0.131). A total of three patients had sudden death in nonsurgery group, and there was no death in surgery group (*P=*0.180). Five patients (5/12, 41.7%) still remain the low heart failure (LVEF ≤40%) after 6 months of enrollment only given medical therapy, and none of patients present with LVEF ≤40% in PAB surgery group (0/6, 0% vs. 8/12, 67.7%, *P=*0.034).

**Conclusion:**

Pulmonary artery banding is safe and effective in infants and young children with end-stage DCM with preserved right ventricular function. Combined with conventional heart failure medication therapy, it may provide short-term benefits postoperatively, decrease the cardiogenic shock, act as a bridge to recovery, and potentially reduce the need for heart transplantation. Long-term effects remain further observation, and larger randomized controlled trials would be more persuasive in validating its efficacy.

## Introduction

Highlights

- Pulmonary artery banding is safe and effective in infants and children with end-stage DCM with preserved right ventricular function.
- Pulmonary artery banding combined with medication therapy could restore LV function, decrease heart shock.
- Pulmonary artery banding would delay or avoid heart transplantation.

Pediatric dilated cardiomyopathy (DCM) is a myocardial disorder characterized by left ventricular dilation and systolic dysfunction, with an annual incidence of 0.57 per 100 000 in children younger than 18 years^1^. The median age at diagnosis was 1.5 years, and age of high accidence was younger than 1 year^1^. Diagnosis of DCM in infants and young children is often delayed for atypical symptoms, and clinical presentation varies from asymptomatic, poor feeding, respiratory distress, and growth failure. Other life-threatening risks including ventricular arrhythmias and atrioventricular block, syncope, cardiogenic shock, and sudden death^1^. More than 80% individuals reach a diagnosis when the patient is state of end-stage sever heart failure^2^. Although 20–45% of patients regain normal cardiac function^3^, the outcome after presentation with DCM remains poor for only 60% transplant-free survival rates within 5 years^4^. And it is summarized that the 1-year and 5-year rates of death or transplantation were 31 and 46%^1^.

The purpose of treating DCM is to relieve acute and chronic heart failure symptoms, postpone the disease progression, and decrease the related complications. It is uncertain whether children with heart failure (HF) could benefit from medical treatment. Studies of HF medication therapy in pediatric DCM are limited, only a small portion of drugs can be used to treat heart failure in children^5,6^. Some of studies have suggested that given immunosuppressive therapy consisting of intravenous immunoglobulin G and prednisolone could benefit ventricular function^7,8^. The indications of immunosuppressive therapy, duration of administration, and efficacy are still unknown. Heart transplantation is the treatment of choice for children with end-stage HF who remain symptomatic despite optimal medical therapy. Although the survival rate after transplantation is satisfied (1 year= 91.5%, 5 year= 83%)^9^, pediatric cardiac transplant is still limited with the high cost, shortage of donors, and series of complications.

Pulmonary artery banding (PAB), a new indication for an old technique, is used to treatment the end-stage left ventricular DCM with preserved right ventricular function. Some pediatric heart centers are performing this kind of procedure for possible myocardial remodeling to improve cardiac function. Currently, the effectiveness of PAB surgical treatment is unclear. There are no corresponding data for PAB surgery combined with medication therapy could improve prognosis in pediatric DCM, comparing with only medication therapy.

This study aimed to investigate the clinical outcomes of PAB surgery in a pediatric population with DCM with preserved right ventricular function. The patient survival and change of cardiac function were systematically compared.

## Methods

### Study population and design

We conducted a retrospective, single-center study. This study was approved by the our Hospital Institutional Review Board. This work has been reported in line with the strengthening the reporting of cohort, cross-sectional, and case–control studies in surgery (STROCSS) criteria^10^.

We enrolled pediatric patients under 5-years-old with end-stage DCM who’s left ventricular function (LVEF≤30%) and right ventricular function (RVEF) were normal, between 1 September 2021 and 31 December 2023 in Beijing Fuwai Hospital. The diagnosis of DCM^1,6^ was based on clinical manifestation, gene test, echocardiographic imaging, and cardiac MRI. The criteria for inclusion were as follows: (1) age ≤5 year old; (2) diagnosis of DCM; (3)LVEF ≤30%; (4) RVEF ≥45%; (5) informed consent and voluntary participation; and (6) complete clinical data. The exclusion criteria were as follows: (1) age >5 year old; (2)ischemic disease, valvular disease, coronary disease, myocarditis, metabolic diseases, and autoimmune disease; (3)LVEF >30%; (4) RVEF <45%; (5) unwilling to participate in study; (6) Pulmonary hypertension out of proportion with left-ventricular end-stage cardiomyopathy; and (7) incomplete clinical data.

All DCM patients received medication treatment for the first 2 months. After drug treatment, seven patients were excluded (five improved heart function (LVEF ≥40%), two lost follow-up). For the remaining patients, they were divided into surgical and nonsurgical group based on whether they underwent PAB surgery. Both groups received continued medication treatment in the follow-up period.

Patients were regularly followed-up by our cardiac ultrasound, with all relevant data recorded. The follow-up duration was 1, 3, and 6 months after enrollment. During this time frame, we compared the short clinical outcomes, including death and change of cardiac function between the groups.

### Medication treatment of DCM

All patients received optimal medication treatment according to guideline-recommended HF therapy^5,6^, including beta-adrenergic blocking agent, angiotensin converting enzyme-inhibitor, aldosterone-antagonist, loop diuretics, digoxin, and nutrients therapy (i.e. co-enzyme Q, carditin, and fructose sodium diphosphate). Dosage of therapy is adjusted with heart rates, blood pressure, urine output, and degree of heart failure. Patients were given the immunosuppressive agents at least one cycle of immunoglobulin and 2 months cycle of prednisone^7,8^.

### Pulmonary artery banding

All operations were performed by one experienced surgeon. The surgery was performed through a median sternotomy incision. The band, made of polytetrafluoroethylene, was placed around the pulmonary arterial trunk, fixed with prolene 6.0. The pulmonary artery circumference could be reduced to about 50–60% of the original diameter. The pulmonary valve and bifurcation of the pulmonary artery should not be bothered by the band. The right ventricular pressure and aortic pressure were detected intraoperatively to adjust the band tightness. The tightness of the band was determined by the systolic RVP/SAP ratio (0.5–0.6).

### Data collection

Baseline demographic characteristics (i.e. age, sex, weight, height, and BMI) was collected at enrollment. Clinical data (i.e. alanine aminotransferase, aspartate aminotransferase, total bilirubin, creatinine, uric acid, N-terminal fragment pro-brain natriuretic peptide, high sensitivity-cardiac troponin T, creatine kinase MB, and genetic abnormalities), use of pharmacological HF treatment, and echocardiographic measurement (i.e. LVDd, LVDd z-score, LVDs, LVDs z-score, LVEF, pulmonary hypertension, tricuspid valve regurgitation, and mitral valve regurgitation) were collected at enrollment and during annual follow-up visits.

### Outcome measurements

The primary endpoint was recovery of ventricular function. The secondary endpoints were 180-day mortality and severe heart failure (LVEF ≤30%). To estimate left ventricular function, left ventricular end-diastolic diameter (LVDd), left ventricular end-systolic diameter (LVDs), and LVEF were calculated at 1, 3, and 6 months. And PAB pressure gradient was calculated to evaluate RV afterload at 1, 3, and 6 months.

### Statistical analysis

The categorical variables were represented as frequencies and percentages. Continuous variables were represented as medians with interquartile ranges (IQRs). The Mann–Whitney *U* test was used to compare continuous variables. The *χ* ^2^ test and Fisher’s exact test were used to compare categorical variables. All statistical tests were two-tailed and a *P*-value <0.05 was considered statistically significant. All data were analyzed using SPSS 26.0. Statistical figures were made using GraphPad Prism 8.0.

## Results

### Study cohort

**Figure 1:**
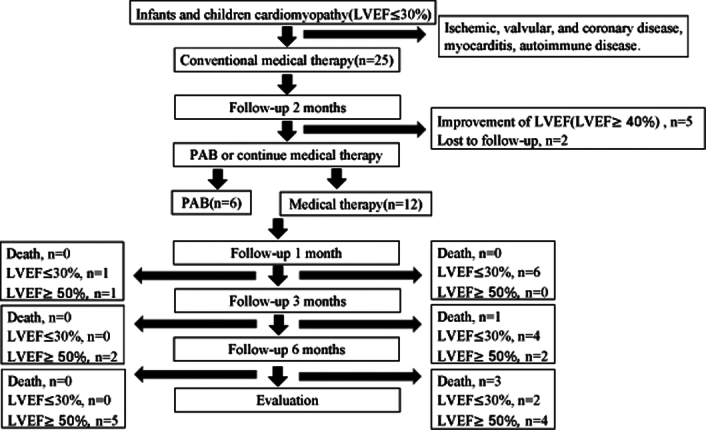
Cohort selection criteria and study flow. Flowchart depicting the inclusion criteria and short-term. outcomes. A total of 18 patients were randomized to surgery or nonsurgery group. LVEF, left ventricular function; PAB, pulmonary artery banding.

We initially identified 25 patients who diagnosed of DCM (LVEF ≤30%, RVEF was normal) in our center between September 2019 and December 2023 (Fig. 1). All patients were given 2-month drug treatment. And then seven patients were excluded due to improvement of LVEF (≥40%) (*n*=5), and no follow-up data (*n*=2). Among the remaining patients, 18 patients were divided into the surgery group (*n*=6) and nonsurgery group (*n*=12) based on whether they underwent PAB surgery.

### Baseline demographic characteristics

In our study, patient sex [5(83.33%) vs. 4(33.33%), *P*=0.131], age [8.00(4.00, 35.00) vs. 8.00(5.25, 14.50), *P*=0.621], weight [6.25(6.00, 9.88) vs. 8.40 (6.40, 9.48), *P*=0.498], height [66.00 (62.25, 85.75) vs. 70.00 (66.00, 79.50), *P*=0.854], and BMI [14.47(13.18, 15.70) vs. 15.42(14.70, 16.62), *P*=0.124] were comparable between surgery group and nonsurgery group. There were no statistical difference in clinical characteristics including ALT [18.41(12.50, 25.75) vs. 16.50 (12.50, 24.00), *P*=0.964], AST [26.00(17.25, 36.00) vs. 43.00(30.00, 46.50), *P*=0.067], TBil [7.53(6.22, 8.89) vs. 5.58(3.68, 8.34), *P*=0.250], Cr [24.05(17.10, 35.87) vs. 25.60(21.23, 32.92), *P*=0.750], Uric [286.75(248.49, 371.75) vs. 305.65(259.04,391.75), *P*=0.964], NT-proBNP [5999.50(880.75, 16320.25) vs. 4424.00(952.00, 30351.00), *P*=0.964], hs-cTnT [0.03(0.02, 0.04) vs. 0.06(0.02, 0.09), *P*=0.335], CK-MB [3.16(2.10, 3.60) vs. 3.49(2.10, 5.63), *P*=0.494], and genetic abnormalities (Table 1). Similar enrollment status of echocardiographic parameters was observed between the surgery group and nonsurgery group, including LVDd, LVDd-Z score, LVDs, LVDs-Z score, LVEF, pulmonary hypertension, tricuspid valve regurgitation, and mitral valve regurgitation. Patients in the surgery group had a higher LVDd-Z score than those in the nonsurgery group [11.70(9.28, 37.11) vs. 8.71(6.45, 9.93), *P*=0.024] (Table 1).

**Table 1:** Baseline demographics.

	Surgery group (*n*=6)	Nonsurgery group (*n*=12)	*P*
Sex (male)	5 (83.33%)	4 (33.33%)	0.131
Age (months)	8.00 (4.00–35.00)	8.00 (5.25–14.50)	0.621
Weight (kg)	6.25 (6.00–9.88)	8.40 (6.40–9.48)	0.498
Height (cm)	66.00 (62.25–85.75)	70.00 (66.00–79.50)	0.854
BMI (kg/m^2^)	14.47 (13.18–15.70)	15.42 (14.70–16.62)	0.124
ALT (IU/l)	18.41 (12.50–25.75)	16.50 (12.50–24.00)	0.964
AST (IU/l)	26.00 (17.25–36.00)	43.00 (30.00–46.50)	0.067
TBil (µmol/l)	7.53 (6.22–8.89)	5.58 (3.68–8.34)	0.250
Cr (umol/l)	24.05 (17.10–35.87)	25.60 (21.23–32.92)	0.750
Uric (umol/l)	286.75 (248.49–371.75)	305.65 (259.04–391.75)	0.964
NT-proBNP (pg/ml)	5999.50 (880.75–16320.25)	4424.00 (952.00–30351.00)	0.964
hs-cTnT (ng/ml)	0.03 (0.02–0.04)	0.06 (0.02–0.09)	0.335
CK-MB (ng/ml)	3.16 (2.10–3.60)	3.49 (2.10–5.63)	0.494
Genetic abnormalities			
TNNT2	2 (33.3%)	4 (33.3%)	1.000
DMD	1 (16.7%)	2 (16.7%)	1.000
TNNI3	0 (0)	1 (8.3%)	0.467
Echocardiography			
LVDd (mm)	45.00 (40.75–55.00)	42.50 (34.00–47.25)	0.291
LVDd-Z score	11.70 (9.28–37.11)	8.71 (6.45–9.93)	0.024
LVDs (mm)	39.50 (36.25–49.00)	38.50 (31.00–40.75)	0.385
LVDs-Z score	16.33 (14.83–20.73)	14.84 (12.05–16.59)	0.125
LVEF (%)	25.50 (18.75–30.00)	25.00 (23.00–30.00)	0.682
Pulmonary hypertension (mmHg)	15.5 (14.3–19.0)	15.0 (13.3–18.0)	0.616
Tricuspid valve regurgitation (n)			
Light	4 (66.7%)	7 (58.3%)	0.732
Moderate-severe	0	0	–
Mitral valve regurgitation (n)			
Light	3 (50.0%)	7 (58.3%)	0.737
Moderate	3 (50.0%)	5 (41.7%)	0.737

Demographic and clinical characteristics of diagnosis of heart failure with DCM stratified by PAB surgery group and nonsurgery group.

Data are presented as *n* (%) or median (interquartile range).

ALT, alanine aminotransferase; AST, aspartate aminotransferase; CK-MB, creatine kinase MB; Cr, creatinine; hs-cTnT, high sensitivity-cardiac troponin T; NT-proBNP, N-terminal fragment pro-brain natriuretic peptide; TBil, total bilirubin; Uric, uric acid.

### Medical management

All patients were given optimal medication treatment in the form of multidrug combinations. Medication therapy was regulated to the maximum tolerated dosage for each patient, based on heart rate, blood pressure, urine output, edema, and heart function. There is no statistical difference in terms of drug use, including beta-adrenergic blocking agent [4(66.7%) vs. 5(41.7%), *P*=0.317], ACE-inhibitor [5(83.3%) vs. 11(91.7%), *P*=0.596], aldosterone-antagonist [6(100%) vs. 11(91.7%), *P*=0.467], loop diuretics [6(100%) vs. 12(100%)], digoxin [5(83.3%) vs. 10(83.3%), *P*=1.000], supplemental therapy [4(66.7%) vs. 8(66.7%), *P*=1.000] (Table 2).

**Table 2:** Phamacological HF treatment in infants and children with DCM.

	Surgery group (*n*=6)	Nonsurgery group (*n*=12)	*P*
Beta-adrenergic blocking agent, n	4 (66.7%)	5 (41.7%)	0.317
ACE-inhibitor, n	5 (83.3%)	11 (91.7%)	0.596
Aldosterone-antagonist, n	6 (100%)	11 (91.7%)	0.467
Loop diuretics, n	6 (100%)	12 (100%)	–
Digoxin, n	5 (83.3%)	10 (83.3%)	1.000
Nutrients, n	4 (66.7%)	8 (66.7%)	1.000

Data are presented as n (%).

ACE, angiotensin converting enzyme; Nutrients, co-enzyme Q, carditin, or fructose sodium diphosphate.

### Heart function

LVEF of survival patients increased during the study between surgery group and nonsurgery group at 3 months [44.00(42.00, 54.00) vs. 35.00(25.00, 43.00), *P*=0.078] and 6 months [56.50(50.25, 66.00) vs. 40.00(36.00, 60.50), *P*=0.113]. The greater increase in LVEF with surgery group was observed, although statistical significance was not reached (Table 3).

**Table 3:** Echocardiographic parameters at time point of different periods.

	Surgery group (*n*=6)	Nonsurgery group (*n*=12)	*P*
Follow-up 1 month			
LVDd (mm)	36.50 (33.25–44.75)	38.50 (31.00–46.75)	1.000
LVDd-Z score	6.10 (4.52–8.79)	6.90 (4.25–9.41)	0.964
LVDs (mm)	29.00 (28.00– 38.00)	33.50 (26.50–40.50)	0.750
LVDs-Z score	9.52 (9.44–12.86)	12.23 (5.26–14.78)	1.000
LVEF (%)	32.50 (31.50–40.25)	30.00 (24.50–36.50)	0.291
Follow-up 3 months			
LVDd (mm)	34.50 (30.00–50.50)	40.00 (30.00–45.00)	1.000
LVDd-Z score	4.59 (2.67–10.99)	6.33 (2.89–9.17)	0.733
LVDs (mm)	28.50 (23.50–37.25)	36.00 (25.00–40.00)	0.733
LVDs-Z score	8.62 (5.55–12.29)	10.69 (4.81–14.97)	0.884
LVEF (%)	44.00 (42.00–54.00)	35.00 (25.00–43.00)	0.078
Follow-up 6 months			
LVDd (mm)	32.50 (24.00–47.75)	31.00 (29.00–41.00)	0.955
LVDd-Z score	2.54 (−2.08, 8.34)	1.25 (0.38–5.95)	0.955
LVDs (mm)	25.00 (15.00–35.50)	26.00 (20.00–36.50)	0.689
LVDs-Z score	5.26 (−1.58, 10.02)	5.32 (1.80–12.16)	0.776
LVEF (%)	56.50 (50.25–66.00)	40.00 (36.00–60.50)	0.113

Data are presented as *n* (%) or median (interquartile range).

LVDd, left ventricular end-diastolic diameter; LVDs, left ventricular end-systolic diameter; LVEF, left ventricular ejection fraction.

**Figure 2:**
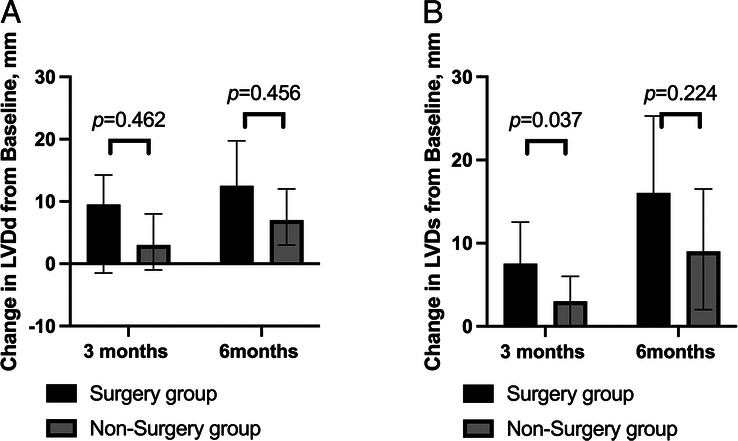
LVDd (A) and LVDs (B) decreased from baseline to 3 and 6 months in patients. LVDd, left ventricular end-diastolic diameter; LVDs, left ventricular end-systolic diameter.

LVDd volume decreased from baseline in the surgery group and nonsurgery group at 3 months [9.50(−1.50, 14.25) vs. 3.00(−1.00, 8.00), *P*=0.462], and at 6 months [12.5(1.25, 19.75) vs. 7.00(3.00, 12.00), *P*=0.456]. LVDs volume decreased from baseline in the surgery group and nonsurgery group at 3 months [7.50(4.25, 12.50) vs. 3.00(0.00, 6.00), *P*=0.037], at 6 months [16.0(5.25, 25.25) vs. 9.00(2.00, 16.50), *P*=0.224]. LVDd from baseline had a larger reduction in the surgery group than the nonsurgery group at 3 months (*P*=0.037) (Fig. 2).

### Patients short-outcomes

**Figure 3:**
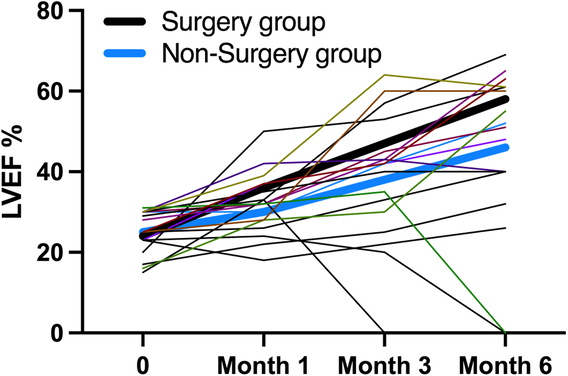
Change of clinical outcome and cardiac function. LVEF, left ventricular ejection fraction.

By the end of the follow-up, there were three (25.0%) deaths in the nonsurgery group, and no one died in the surgical group(*P*=0.515). The survival rates at 1, 3, and 6 months in the surgery group (100%, 100%, and 100%) and nonsurgery group (100%, 91.7%, and 75%) (Fig. 3).

A total of five (83.3%) patients regain the recovery of heart function (LVEF ≥ 50%) in the surgery group, and 4 (33.3%) patients in the nonsurgery group (*P*=0.515). In the nonsurgery group, there were still 2 two (16.7%) patients accompanied with severe heart failure (LVEF ≤30%) and three (25%) patients presenting with low cardiac function (30% ≤LVEF ≤40%). Comparing with surgery group, a total of eight patients had poor prognosis in nonsurgery group [including death (3), severe heart failure (2), and low cardiac function (3)] (*P*=0.013)(Table 4).

**Table 4:** Short clinical outcomes in infants and children with DCM.

	Surgery group (*n*=6)	Nonsurgery group (*n*=12)	*P*
Death, n	0 (0)	3 (25.0%)	0.515
LVEF ≤30%, n	0 (0)	2 (16.7%)	0.529
Recovery of LVEF ( ≥ 50%), n	5 (83.3%)	4 (33.3%)	0.131

Data are presented as *n* (%).

LVEF, left ventricular ejection fraction.

### PAB pressure gradient

**Figure 4:**
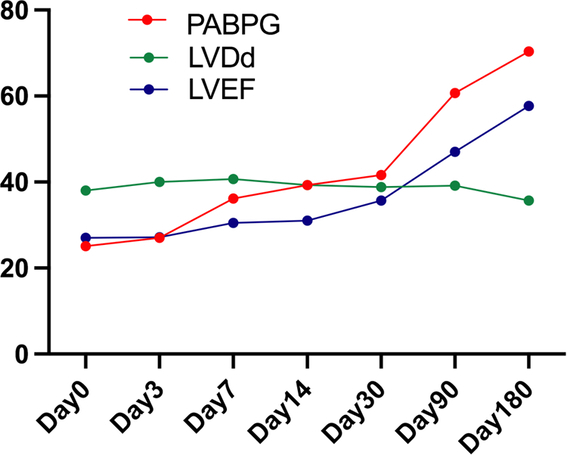
Change of the PAB pressure gradient, LVDd and LVEF in surgery group. LVDd, left ventricular end-diastolic diameter; LVEF, left ventricular ejection fraction; PABPG, pulmonary artery banding pressure gradient.

In the surgery group, a reduction in left ventricular end-diastolic diameter and left ventricular end-systolic diameter were observed within 2 weeks after surgery, but no significant changes in left heart function were observed, and PAB pressure gradient was between 20 and 30 mmHg. A large increase in PAB pressure gradient and left heart function from baseline was observed postoperative 1 month to 3 months. PAB pressure gradient increased to 40–60 mmHg, and LVEF began to improve accompanying with decreased LVDd (Fig. 4).

### Further follow-up in PAB

Based on our long-term follow-up, five patients in the surgery group received recovery in their clinical and hemodynamic status, and three patients were performed a balloon dilatation by transcatheter between 6 months to 1 year after PAB surgery. Only one patient had improvement in cardiac function (LVEF:51%). And they were given the small dosage medication therapy for a possible decline in heart function till now. In nonsurgery group, two patients died between 6 months to 1 year of enrollment. Six received recovery and one still had low cardiac function (LVEF:35%).

## Discussion

Dilated cardiomyopathy is a common cardiomyopathy with the unique features of cardiac dilatation and subnormal to poor myocardial contractility, and become the leading cause of cardiac death in children. When medication therapy is ineffective, mechanical support devices and heart transplantation would be used to treat children with end-stage DCM. Long waiting, shortage of donors, high cost, and series of complications have limited heart transplant application in children especially for infants or young ones. America has shown 1-year mortality rate was 54% in nontransplant children population^11^. To improve outcomes, or avoid heart transplantation, Schranz *et al*.^12^ proposed an alternative surgical strategy of PAB, as a treatment method in children with DCM, when RV function is preserved. Recently, some of centers worldwide have treated PAB as a rescue strategy, aimed at promoting myocardial recovery^13^. In our retrospective study, we utilized a small cases database to investigate the clinical outcomes of PAB surgery in patients with DCM with preserved RV function, assess whether PAB should be considered for end-stage DCM.

In our study, we enrolled 18 patients, 12 (12/18, 66.7%) received medication therapy, 6 (6/18, 33.3%) underwent additional PAB surgery. In nonsurgery group, three (3/12, 25.0%) patients experienced cardiac death. No directly PAB-related death have been described. No one died in the surgery group, although there was no significant difference for the small sample size. Patients were likely to have a poor prognosis (death, LVEF ≤40%) in conventional medical therapy, comparing with surgery group (0/6, 0%), and the result was statistically significant. The vast majority of patients (5/6, 83.3%) have complete recovery of left heart function in surgery group, and only four patients (4/12, 33.3%) received complete recovery in nonsurgery. Comparing with conventional medication therapy, the proportion of cardiac function recovery in the PAB surgery group was significantly increased within 6 months of follow-up. Limited by the small cases of PAB surgery, there is a lack of controlled studies of PAB surgery versus medical treatment alone. The timing of the intervention was also important, and a possible cause of PAB failure is the age of patients. Matteo *et al*.^13^ suggested maximal threshold was 1 year old. Schranz *et al*.^12^ showed the age limit for surgery was 6-year-old age. In our experience, our inclusion criterion was less than 5 years of age. We also found that children less 1 year old had more rapid cardiac improvement.

A multicenter international study^14^ enrolling 61 patients, mean age was 266±310 days, who received selective PAB surgery between 2006 and 2017, showed that operative mortality was 0, 42 (68.9%) patients experienced functional improvement, 34 (56%) experienced functional recovery, 27 (44.3%) experienced pulmonary debanding. We thought that difference in HF etiologies and preoperative status contributed to the difference in recovery rate. In our study, only one patient aged 44 months had functional improvement but not recovery. Poor preoperative status and older age lead to a poor prognosis. Although Schranz *et al*.^12^ suggested age limit for PAB is 6-year-old, we still believed that infants could benefit from surgery with lower age. Matteo *et al*.^13,15^ indicated that the age is the key to PAB success for the repair ability and regenerative pathways of the young heart, and considered a age threshold under 1 year of age, cardiomyocyte proliferation tends to be poorer with aging. This might be the reason for delay recovery in our older patient.

Furthermore, we investigated the reason why PAB could improve LVEF in DCM with preserved RV. In our study, we found that left ventricular volume decreased and left ventricular function improved with PAB pressure gradient increasing simultaneously in surgery group. Meanwhile, with long-term echocardiography, the right-shifted ventricular septum gradually shifts to the left ventricle, which would lead to LV reshaping and restoration^14,16^. Schranz *et al*.^12^ thought that PAB induced RV stress, reduces LV preload, improves LV end-diastolic volume, pressure, and LV filling dynamics, enhanced biventricular function. Compared with diuretic usage, long-term and stable reduction of LV preload induced by PAB surgery is more conducive to the improvement of LV function.

## Conclusion

PAB is an alternative surgical strategy in restoring LV function in young children with DCM with preserved RV function. Combined with conventional heart failure medication therapies, it can decrease the cardiogenic shock, and act as a bridge to recovery and potentially reduce the need for heart transplantation.

## Limitations

There were some limitations to be addressed. Our study is retrospective, single-center, and the limited number of patients. Small sample size and lack of reliable data could not demonstrate a superiority of a strategy over another. Future research should be undertaken to stimulate multicenter, greater number of patients. We hope that PAB surgery would be considered as alternative surgical strategy in infants with DCM. Further prospective studies need to focus on biological working and molecular mechanisms of PAB surgery.

## Ethical approval

This study was approved by the Fuwai Hospital Institutional Review Board, Beijing, People’s Republic of China (2022-1798).

## Consent

All patients or parents gave written informed consent.

## Source of funding

Capital health research and development of special fund, China (2022-1-4032).

## Author contribution

M.Z., F.Y., C.Y., and S.J.L.: participated in research design, data collection, data analysis, and paper writing; W.W., K.M., Z.D., Q.L.L., and X.W.: participated in research design and critical revision.

## Conflicts of interest disclosure

The authors declare no conflicts of interest.

## Research registration unique identifying number (UIN)

NCT06039553.

## Guarantor

Shoujun Li.

## Data availability statement

Data sharing are not applicable to this article.

## Provenance and peer review

Not commissioned, externally peer-reviewed.

## Assistance with the study

None.

## Presentation

None.
